# The Effect of the Aerial Part of *Lindera akoensis* on Lipopolysaccharides (LPS)-Induced Nitric Oxide Production in RAW264.7 Cells

**DOI:** 10.3390/ijms14059168

**Published:** 2013-04-26

**Authors:** Chung-Ping Yang, Guan-Jhong Huang, Hui-Chi Huang, Yu-Chang Chen, Chi-I Chang, Sheng-Yang Wang, Hsun-Shuo Chang, Yen-Hsueh Tseng, Shih-Chang Chien, Yueh-Hsiung Kuo

**Affiliations:** 1 Department of Chinese Pharmaceutical Sciences and Chinese Medicine Resources, China Medical University, Taichung 404, Taiwan; E-Mails: u9752005@cmu.edu.tw (C.-P.Y.); gjhuang@mail.cmu.edu.tw (G.-J.H.); hchuang@mail.cmu.edu.tw (H.-C.H.); yuchang@mail.cmu.edu.tw (Y.-C.C.); 2 Department of Biological Science and Technology, National Pingtung University of Science and Technology, Pingtung 912, Taiwan; E-Mail: changchii@mail.npust.edu.tw; 3 Department of Forestry, National Chung Hsing University, Taichung 402, Taiwan; E-Mails: taiwanfir@dragon.nchu.edu.tw (S.-Y.W.); tseng2005@nchu.edu.tw (Y.-H.T.); 4 Agricultural Biotechnology Research Center, Academia Sinica, Taipei 115, Taiwan; 5 Graduate Institute of Natural Products, Kaohsiung Medical University, Kaohsiung 807, Taiwan; E-Mail: hschang@kmu.edu.tw; 6 The Experimental Forest Management Office, National Chung-Hsing University, Taichung 402, Taiwan; 7 Tsuzuki Institute for Traditional Medicine, China Medical University, Taichung 402, Taiwan

**Keywords:** Chinese herb, *Lindera akoensis*, butanolide, lignans, flavonoids, anti-inflammatory

## Abstract

Four new secondary metabolites, 3α-((*E*)-Dodec-1-enyl)-4β-hydroxy-5β-methyldihydrofuran-2-one (**1**), linderinol (**6**), 4′-*O*-methylkaempferol 3-*O*-α-l-(4″-*E*-*p*-coumaroyl)rhamnoside (**11**) and kaempferol 3-*O*-α-l-(4″-*Z*-*p-*coumaroyl) rhamnoside (**12**) with eleven known compounds—3-epilistenolide D_1_ (**2**), 3-epilistenolide D_2_ (**3**), (3*Z*,4α,5β)-3-(dodec-11-ynylidene)-4-hydroxy-5-methylbutanolide (**4**), (3*E*,4β,5β)-3-(dodec-11-ynylidene)-4-hydroxy-5-methylbutanolide (**5**), matairesinol (**7**), syringaresinol (**8**), (+)-pinoresinol (**9**), salicifoliol (**10**), 4″-*p*-coumaroylafzelin (**13**), catechin (**14**) and epicatechin (**15**)—were first isolated from the aerial part of *Lindera akoensis.* Their structures were determined by detailed analysis of 1D- and 2D-NMR spectroscopic data. All of the compounds isolated from *Lindera akoensis* showed that *in vitro* anti-inflammatory activity decreases the LPS-stimulated production of nitric oxide (NO) in RAW 264.7 cell, with IC_50_ values of 4.1–413.8 μM.

## 1. Introduction

*Lindera akoensis* (Lauraceae) is an endemic evergreen tree that grows in broad-leaved forests in lowlands throughout Taiwan; it is often used as a fence. Aporphines [1], alkaloids [2], sesquiterpenoids [3–5], flavonoids [6], butanolides [6], furanoids [7], chalconoids [8] and phenolic compounds [9,10] are widely distributed in the plants of the genus of *Lindera*. Some isolates exhibit biological activities, including suppressed the contraction of thoracic aorta [1], anti-mycobacterial [6], anti-inflammatory [11], against human lung cancer cell (SBC-3) [12], inhibitory osteoclast differentiation [10], slowing down of the progression of diabetic nephropathy in mice [12], anti-nociceptivity [13], inhibition on human acyl-coenzyme A cholesterol acyltransferase activity and antioxidation of low density lipoprotein [9]. Only one piece of literature had reported the chemical constituents and anti-mycobacterial activity from the root of *L. akoensis* [6].

The folk usage of *L. akoensis* is in the treatment of trauma and inflammation [14]. Butanolides showed anti-inflammation in previous studies [15,16]. In a random screening for inhibitory activity of various Chinese traditional medicines toward nitric oxide (NO) production *in vitro* by RAW264.7 cells, the EtOH extract of the aerial parts of *L. akoensis* showed a significant activity. Thus, the constituents of *L. akoensis* were investigated. This paper deals with the structure elucidation of the new compounds, and the inhibitory activity of the isolates toward nitric oxide (NO) production towards RAW264.7 cells is also discussed.

## 2. Results and Discussion

### Isolation and Structural Elucidation

**Figure 1:**
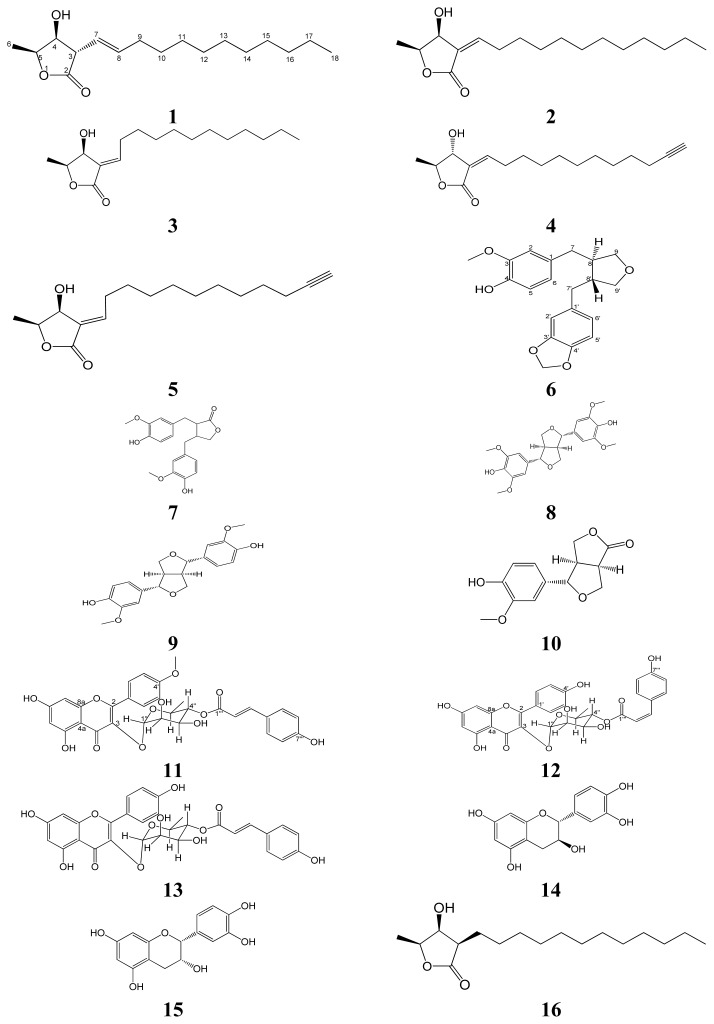
Structures of **1**–**16**.

The aerial part of *L. akoensis* was air-dried and then extracted by EtOH and purified. Extensive normal phase Si gel column chromatographic purification of the EtOAc-soluble fraction afforded four new compounds, 3α-((*E*)-Dodec-1-enyl)-4β-hydroxy-5β-methyldihydrofuran-2-one (**1**), linderinol (**6**), 4′-*O*-methylkaempferol 3-*O*-α-l-(4″-*E*-*p*-coumaroyl)rhamnoside (**11**), kaempferol 3-*O*-α-l-(4″-*Z*-*p-*coumaroyl) rhamnoside (**12**), as well as eleven known compounds, 3-epilistenolide D_1_ (**2**) [17], 3-epilistenolide D_2_ (**3**) [17], (3*Z*,4α,5β)-3-(dodec-11-ynylidene)-4-hydroxy-5-methylbutanolide (**4**) [18], (3*E*,4β,5β)-3-(dodec-11-ynylidene)-4-hydroxy-5-methylbutanolide (**5**) [19], matairesinol (**7**) [20], syringaresinol (**8**) [21], (+)-pinoresinol (**9**) [22], salicifoliol (**10**) [23], 4″-*p*-coumaroylafzelin (**13**) [24], catechin (**14**) [25] and epicatechin (**15**) [25] (Figure 1).

**Figure 2:**
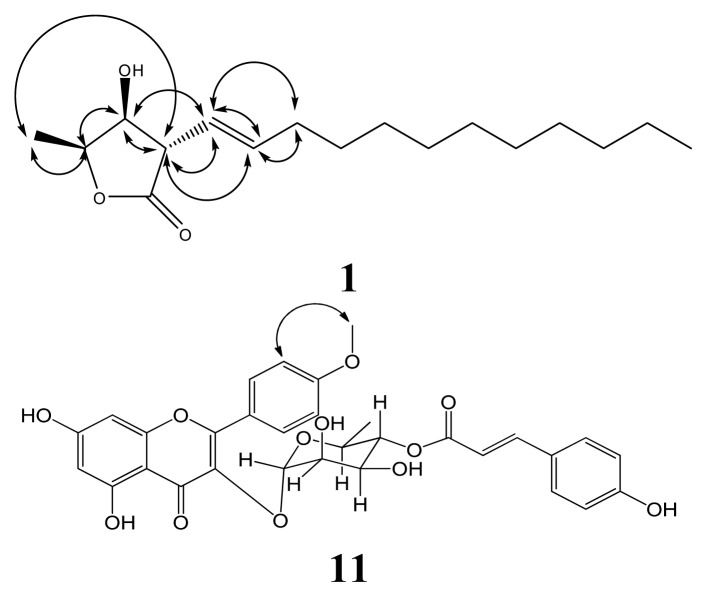
Significant NOESY correlations (↔) of **1** and **11**.

Compound **1** was isolated as an optically inactive colorless oil ([α]^2^°_D_ ± 0 (*c* 0.4, CHCl_3_)) and showed the presence of hydroxy (3401 cm^−1^), olefin (1682 cm^−1^) and γ-lactone (1759 cm^−1^) functionalities groups in its infrared (IR) spectrum. The high resolution electron impact mass spectrometry (HREIMS) data determined the molecular formula to be C_17_H_30_O_3_ (*m/z* 282.2198 ([M]^+^; calcd 282.2195)). The ^1^H-NMR spectrum showed signals similar to those of (3β,4β,5β)-3-dodecyl-4- hydroxy-5-methyldihydrofuran-2-one (**16**) (not purified in this research) [18] at δ_H_ 3.19 (1H, *dd*, *J* = 6.5, 4.7 Hz), δ_H_ 4.23 (*dd*, *J* = 4.7, 4.5), δ_H_ 4.64 (*qd*, *J* = 6.5, 4.5) were assigned to H-3, H-4 and H-5, respectively (Table 1). The chemical shift and coupling patterns of H-4 and H-5 suggested that the relative configuration of **1** was identified similar to that of **16**. This conclusion was supported by comparison of the ^1^H and ^13^C NMR data of **1** with those of reported compounds having a *cis*-relationship between H-4 and H-5. ^1^H-NMR spectrum of **1** was similar to that of compounds **3** with 4β-hydroxy-5β-methyl groups. Two olefinic H-atoms were assigned the signals at δ_H_ 5.37 (1H, *dd*, *J* = 15.4, 6.5 Hz, H-7), δ_H_ 5.72(1H, *dt*, *J* = 15.4, 7.4 Hz, H-8), and nine CH_2_-group signals were observed (δ_H_ 2.04 (2H, *q*, *J* = 7.4 Hz, H-9), δ_H_ 1.24 (16H, m, H-10–17)). The H-7 was coupled with H-3 and H-8, with coupling constant 6.5 Hz and 15.4 Hz, respectively, establishing the *trans*-geometry of Δ^7^. Compared with 3β-((*E*)-dodec-1-enyl)-4β-hydroxy-5β-methyldihydrofuran-2-one in our previous study [26], the only difference was the configuration of the H-3((δ_H_ 3.19, *dd*, *J* = 6.5, 4.7 Hz), δ_C_ 52.7 (C-3)). The key correlation of NOESY spectrum, H-3, has correlation with H-6 and no correlation with H-5, moreover H-4 and H-5 having NOESY correlation, confirmed that H-4 and H-5 in the same phase and H-3 was in opposite side of H-3 (Figure 2). The zero optical rotation value indicated that there may exist in compound **1** a racemic mixture. All protons and carbons were confirmed by 1D and 2D spectra. Thus, **1** was identified as 3α-((*E*)-dodec-1-enyl)-4β-hydroxy-5β-methyldihydrofuran-2-one.

Compound **6** was a pale yellow amorphous solid, ([α]^2^°_D_ = +20.2° (c = 0.42, CHCl_3_)); it has a λ_max_ at 284.6 nm (logɛ 3.27) in the ultraviolet (UV) spectrum and shows the presence of hydroxy (3310 cm^−1^) and benzene (1605 and 1512 cm^−1^) functionalities in its IR spectrum. The HREIMS data determined the molecular formula to be C_20_H_22_O_5_ (*m/z* 342.1530 ([M]^+^; calcd 342.1467)). The ^1^H-NMR spectrum showed signals similar to those of matairesinol (**7**), such as the CH_2_ group at δ_H_ 2.62 (2H, *dd*, *J* = 12.8, 5.0 Hz, Ha-7, Ha-7′), δ_H_ 2.72 (2H, *dd*, *J* = 12.8, 8.5 Hz, Hb-7, Hb-7′), δ_H_ 3.51 (2H, *dd*, *J* = 11.2, 6.4 Hz, Ha-9, Ha-9′), δ_H_ 3.77 (2H, *dd*, *J* = 11.2, 6.4 Hz, Hb-9, Hb-9′) were assigned to H-7, H-7′, H-9 and H-9′, respectively. One set of the ABX system of aromatic protons exhibited at δ_H_ 6.61(1H,1H, *s*, H-2), δ_H_ 6.68 and 6.79 (each 1H, *d*, *J* = 8.0 Hz, H-6, H-5); the other set of aromatic protons showed at δ_H_ 6.61 (1H, *s*, H-2′), δ_H_ 6.57 and 6.62 (each 1H, *d*, *J* = 8.0 Hz, H-6′, H-5′). The proton signals assignments are elucidation by HMBC technology. In addition, three functional groups attached on the different phenyl groups were revealed from the following ^1^H-NMR signals: δ_H_ 5.90 (2H, *s*, methylene dioxide), 3.82 (3H, Ar-OMe, having a NOESY correlation to H-2) and 5.66(1H, *s*, Ar-OH). The positive value of optical rotation could be inferred the *trans*-configuration between dibenzyl substituents on C-8 and C-8′. Based on the ^1^H- and ^13^C-NMR (Table 1), COSY, NOESY, HSQC and HMBC experiments, the structure of **6** was tentatively named as linderinol.

Compound **11** was a pale yellow amorphous solid, ([α]^2^°_D_ ± 0° (c = 8.3, CH_3_OH)). Its molecular formula was determined to be C_31_H_28_O_12_ by HR-ESI-MS spectrometry (*m/z* 592.1576 ([Na]^+^; calcd 592.5446). The IR spectrum exhibited bands at 3426 and 1651 cm^−1^ due to a hydroxyl and a conjugated carbonyl group. The NMR signals of rhamnose were easily assigned by their characteristic multiplicities, especially on the unique proton signal of the methyl, which was up-field at δ_H_ 0.78 (3H, *d*, *J* = 6.3 Hz), shielded by a C-ring, the aromatic ring of flavon [24]. An A_2_X_2_ coupling system at δ_H_ 7.49 (2H, *d*, *J* = 8.6 Hz, H-5‴, -9‴) and 6.84 (2H, *d*, *J* = 8.6 Hz, H-6‴, -8‴), as well as two olefinic proton signals at δ_H_ 6.25 and 7.53 (each 1H, *d*, *J* = 16.0 Hz) could be observed in the presence of a *E*-*p*-coumaroyl moiety. The H-4″ triplets (δ_H_ 4.91, t, *J* = 9.7 Hz) in this compound appeared at a relatively low field with respect to the corresponding signal of afzelin [24]. Hence, this compound is esterified at this position. The apigenin group could be observed by NMR spectra, matching the literature [27], but the proton signal at H-3 (δ_H_ 6.76, 1H, *s*) cannot be detected; moreover, a conspicuous difference of the carbon signal between C-3 of **11** (δ_C_ 135.7) and C-3 of apigenin (δ_C_ 103.2) was observed. By this evidence, we speculated that rhamnose connected on apigenin with a C-3-C-1″ linkage, just like the common afzelin; this speculation was certificated by 1- and 2-D NMR. A methoxy, with a resonance at δ_H_ 3.85 (3H, *s*), correlated with C-4′ (δ_C_ 163.6) on the HMBC spectrum, indicating that C-4′ was the position where it linked with a methoxy; furthermore, the significant NOE correlation on position 3′ (δ_H_ 7.14, 2H, *d*, *J* = 8.8) and a methoxy (δ_H_ 3.85, 3H, *s*) proved this. The rhamnoside and *E*-*p*-coumaroyl configurations were decided by the 1D-, 2D-NMR and comparison of the ^1^H- and ^13^C-NMR spectrum of compound **13** [24]. Based on the above deduction, **11** was designated to be a new compound 4′-*O*-methylkaempferol 3-*O*-α-l-(4″-*E*-*p*-coumaroyl)rhamnoside.

Compound **12** was a pale yellow amorphous solid, ([α]^2^°_D_ ± 0° (c = 4.5, CH_3_OH)). Its molecular formula was determined to be C_30_H_26_O_12_ by HR-ESI-MS spectrometry (*m/z* 578.1416 ([Na]^+^; calcd 578.1424). Together, a 2D technique predicted **12** as a combination with three units of *p*-coumaroyl, rhamnose and kaempferol derivative, such as in compound **13** [24]; the *Z*-configuration of C-2‴ and C-3‴ on the *p*-coumaroyl moiety was deduced by the smaller coupling constant (12.8 Hz), a higher shift of two olefinic proton signals (δ_H_ 5.75 and 6.87) and a lower shift of H-5‴ (δ_H_ 7.66, *d*, *J* = 8.6 Hz), comparing to the corresponding protons in **11**. Based on the above deduction, **12** was designated as a new compound, kaempferol 3-*O*-α-l-(4″-*Z*-*p*-coumaroyl)rhamnoside.

3-epilistenolide D_1_ (**2**), 3-epilistenolide D_2_ (**3**), (3*Z*,4α,5β)-3-(dodec-11-ynylidene)-4-hydroxy-5- methylbutanolide (**4**) and (3*E*,4β,5β)-3-(dodec-11-ynylidene)-4-hydroxy-5-methylbutanolide (**5**) were isolated as light yellow oils, whereas matairesinol (**7**), syringaresinol (**8**), (+)-pinoresinol (**9**), salicifoliol (**10**), 4″-*p*-coumaroylafzelin (**13**), catechin (**14**) and epicatechin (**15**) were obtained as pale yellow solids. The ^1^H and ^13^C NMR spectra of compounds **2**–**5**, **7**–**10** and **13**–**15** were confirmed by comparison of their spectral data with the reported value from the literature.

### 2.2 Anti-Inflammatory Activity

NO, produced from l-arginine by NO synthase, has various biological actions, e.g., as a defense and regulatory molecule for homeostatic equilibrium [28]. However, in pathophysiologic conditions, such as inflammation, there is an increased production of NO by inducible NO synthase (iNOS) [29]. Macrophages have been expected to be an origin of inflammation, because they contain various chemical mediators that may be responsible for several inflammatory stages [30]. The inhibitory activity toward NO production, induced by lipopolysaccharides (LPS), by murine macrophage-derived RAW264.7 cells, was assayed. These compounds from *L. akoensis* were screened by anti-inflammatory activity *in vitro* with a decrease in nitrite of the LPS-stimulated production in RAW 264.7 cells with IC_50_ values of 4.1–413.8 μM (Table 2).

## 3. Experimental Section

### 3.1. Chemicals

LPS (endotoxin from *Escherichia coli*, serotype 0127:B8), indomethacin, MTT (3-[4,5-dimethylthiazol-2-yl]-2,5-diphenyltetrazolium bromide) and other chemicals were purchased from Sigma Chemical Co. (St. Louis, MO, USA).

### 3.2. General

UV spectra were obtained with a Shimadzu Pharmaspec-1700 UV-Visible spectrophotometer. Optical rotations were obtained with a Jasco P-1020 polarimeter. Infrared spectra were obtained with a Shimadzu IRprestige-21 Fourier transform infrared spectrophotometer. 1D- and 2D-NMR spectra were recorded with a Bruker DRX-400 FT-NMR spectrometer. Mass spectrometric (HR-EI-MS and HR-ESI-MS) data were generated at the Mass Spectrometry Laboratory of the Chung Hsing University. Column chromatography was performed using Merck Si gel (30–65 μM), and TLC analysis was carried out using aluminum pre-coated Si plates; the spots were visualized using a UV lamp at λ = 254 nm. HPLC chromatograms were obtained with a Shimadzu LC-6A and a IOTA-2 RI-detector with a Phenomenex luna silica(2) 250 × 10 column.

### 3.3. Plant Material

*Lindera akoensis* was collected and identified by Dr. Yen-Hsueh Tseng (Department of Forestry, National Chung Hsing University) at Taichung, Taiwan, in July, 2008.

### 3.4. Extraction and Isolation

**Figure 3:**
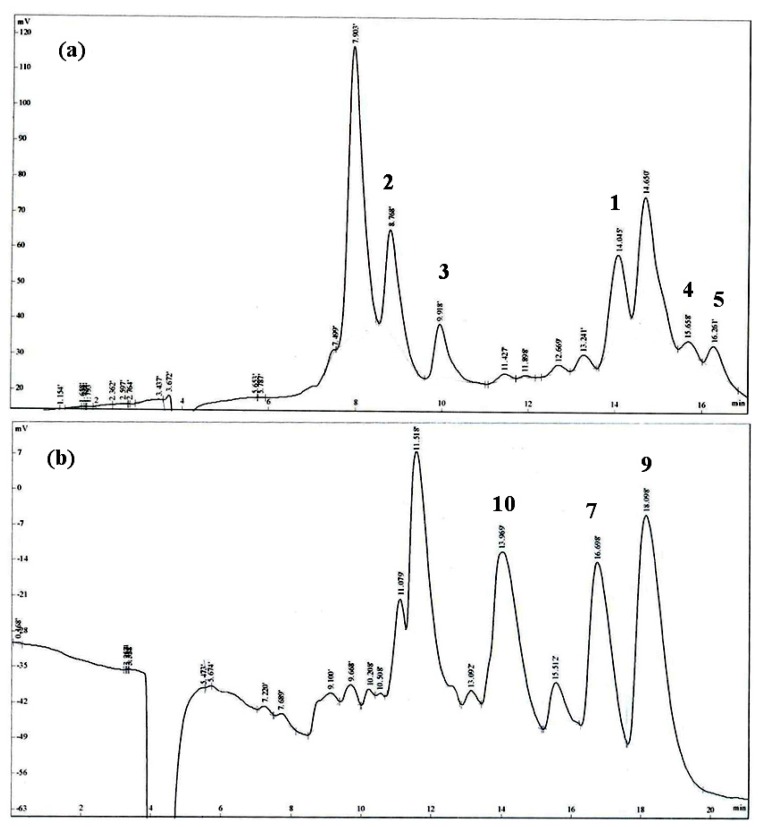

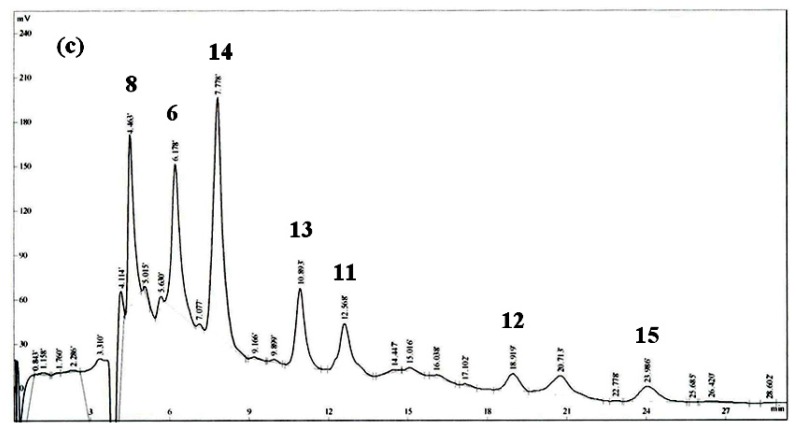
The chromatograms of **1**–**15** on semi-preparative HPLC.

The materials were totally dried under dark in air. The dried aerial part of *L. akoensis* (5.9 kg) was cut into small pieces and soaked in 95% ethanol (60 liter, 7 days × 3). After filtration, the crude extract was concentrated and stored under vacuum to yield an brown thick paste (337.8 g) that was suspended in H_2_O (1000 mL) and extracted with ethyl acetate (1000 mL, 3 times). The resulting ethyl acetate extract was concentrated to yield 127.8 g of a brown thick oil that was purified by 1900 g silica gel with a particle size 0.063–0.200 mm and an internal diameter of the column, 15 cm packed height, 25 cm chromatography, using a gradient of increasing polarity with *n*-hexane/ethyl acetate (99:1–0:100) as the mobile phase and separated into 21 fractions on the basis of TLC analysis for random isolation of compounds. Fraction 11 (5.08 g) was re-separated by chromatography and semi-preparative HPLC with 40% EtOAc in *n*-hexane to afford pure, butanolide **1** (6.1 mg, 0.00104‰), **2** (7.7 mg, 0.00131‰), **3** (7.8 mg, 0.00132‰) and **4** (2.1 mg, 0.00036‰) and **5** (1.6 mg, 0.00027‰). Fraction 15 (6.82 g) was re-separated by chromatography and semi-preparative HPLC with 50% EtOAc in *n*-hexane to afford pure lignans **7** (2.3 mg, 0.00039‰), **9** (1.5 mg, 0.00025‰) and **10** (1.2 mg, 0.00020‰). Fraction 16 (7.15 g) was re-separated by chromatography and semi-preparative HPLC with 60% EtOAc in *n*-hexane to afford pure lignans **6** (8.3 mg, 0.00141‰), **8** (15.8 mg, 0.00268‰), **11** (16.6 mg, 0.00281‰), **12** (8.9 mg, 0.00151‰), **13** (38.3 mg, 0.00649‰), **14** (62.3 mg, 0.01056‰) and **15** (2.2 mg, 0.00037‰). The flow of semi-preparative HPLC was 1.5 mL/min, the chromatograms of compounds showed on Figure 3.

3α-((*E*)-dodec-1-enyl)-4β-hydroxy-5β-methyldihydrofuran-2-one (**1**). Colorless oil; mp: 75.5–77.0 °C; [α]^2^°_D_ ± 0° (*c* = 0.4, CHCl_3_); HR-EI-MS *m/z:* 282.2198 [M]^+^ (calcd for C_17_H_30_O_3_, 282.2195); IR (KBr) λ_max_: 3401, 1759, 1682, 1379 cm^−1; 1^H-NMR and ^13^C-NMR (400/100 MHz, in CDCl_3_): see Table 1.

Linderinol (**6**). Yellow amorphous solid; mp: 76.0–76.5°C; [α]^2^°_D_ + 20.2° (c = 0.42, CHCl_3_); HR-EI-MS *m/z:* 342.1530 [M]^+^ (calcd for C_20_H_22_O_5_, 342.1467); UV_max_ (CH_3_OH): 253, 284 nm; IR (KBr) λ_max_: 3310, 1605, 1512 cm^−1; 1^H-NMR and ^13^C-NMR (500/125 MHz, in CDCl_3_): see Table 1.

4′-*O*-methylkaempferol 3-*O*-α-l-(4″-*E*-*p*-coumaroyl) rhamnoside (**11**). Yellow amorphous solid; [α]^2^°_D_ ±0° (c = 8.3, CH_3_OH); HR-ESI-MS *m/z:* 592.1576 [Na]^+^ (calcd for C_31_H_28_O_12_, 592.5446); UV_max_ (CH_3_OH): 313, 277, 267, 247 nm; IR (KBr) λ_max_: 3426, 2924, 1651, 1605, 1512, 1173 cm^−1; 1^H-NMR and ^13^C-NMR (500/125 MHz, in CDCl_3_): see Table 3.

Kaempferol 3-*O*-α-l-(4″-*Z*-*p*-coumaroyl) rhamnoside (**12**). Yellow amorphous solid; [α]^2^°_D_ ± 0° (c = 4.5, CH_3_OH); HR-ESI-MS *m/z:* 578.1416 [Na]^+^ (calcd for C_30_H_26_O_12_, 578.1424); UV_max_ (CH_3_OH): 313, 277, 266, 247 nm; IR (KBr) λ_max_: 3418, 2978, 1651, 1605, 1513, 1173 cm^−1; 1^H-NMR and ^13^C-NMR (500/125 MHz, in CDCl_3_): see Table 3.

### 3.5. Cell Culture

A murine macrophage cell line RAW264.7 (BCRC No. 60001) was purchased from the Bioresources Collection and Research Center (BCRC) of the Food Industry Research and Development Institute (Hsinchu, Taiwan). Cells were cultured in plastic dishes containing Dulbecco’s Modified Eagle Medium (DMEM, Sigma, St. Louis, MO, USA) supplemented with 10% fetal bovine serum (FBS, Sigma, St. Louis, MO, USA, USA) in a CO_2_ incubator (5% CO_2_ in air) at 37 °C and subcultured every 3 days at a dilution of 1:5 using 0.05% trypsin-0.02% EDTA in Ca^2+^-, Mg^2+^-free phosphate-buffered saline (DPBS).

### 3.6. Measurement of Nitric Oxide/Nitrite

NO production was indirectly assessed by measuring the nitrite levels in the cultured media and serum determined by a colorimetric method based on the Griess reaction. The cells were incubated with butanolides (0, 3.125, 6.25, 12.5, 25 and 50 μg/mL) in the presence of LPS (100 ng/mL) at 37 °C for 24 h. Then, cells were dispensed into 96-well plates, and 100 μL of each supernatant was mixed with the same volume of Griess reagent (1% sulfanilamide, 0.1% naphthyl ethylenediamine dihydrochloride and 5% phosphoric acid) and incubated at room temperature for 10 min; the absorbance was measured at 540 nm with a Micro-Reader (Molecular Devices Orleans Drive, Sunnyvale, CA, USA). Serum samples were diluted four times with distilled water and deproteinized by adding 1/20 volume of zinc sulfate (300 g/L) to a final concentration of 15 g/L. After centrifugation at 10,000× *g* for 5 min at room temperature, 100 μL supernatant was applied to a microtiter plate well, followed by 100 μL of Griess reagent. After 10 min of color development at room temperature, the absorbance was measured at 540 nm with a Micro-Reader. By using sodium nitrite to generate a standard curve, the concentration of nitrite was measured form absorbance at 540 nm.

### 3.7. Cell Viability

Cells (2 × 105) were cultured in 96-well plate containing DMEM supplemented with 10% FBS for 1 day to become nearly confluent. Then cells were cultured with compounds **1**–**5** in the presence of 100 ng/mL LPS (lipopolysaccharide) for 24 h. After that, the cells were washed twice with DPBS and incubated with 100 μL of 0.5 mg/mL MTT for 2 h at 37 °C testing for cell viability. The medium was then discarded, and 100 μL dimethyl sulfoxide (DMSO) was added. After 30-min incubation, the absorbance at 570 nm was read using a microplate reader (Molecular Devices, Sunnyvale, CA, USA).

### 3.8. Statistical Analysis

IC_50_ values were estimated using a non-linear regression algorithm (SigmaPlot 8.0; SPSS Inc., Chicago, IL, USA, 2002). Statistical evaluation was carried out by one-way analysis of variance (ANOVA followed by Scheffe’s multiple range tests).

## 4. Conclusions

These fifteen compounds **1**–**15** exhibited no significant cytotoxic activity. As to anti-inflammatory activity, compounds **2** and **3** are stronger than the other three, butanolides **1**, **4** and **5**. According to our previous study [26], the active site may result from the conjugation between the γ-lactone and olefinic functionalities despite the *E-* or *Z*-form. Although compounds **4** and **5** also possessed conjugation of γ-lactone and olefinic functionalities, it showed no significant active, due to the terminal acetylene group retarding the activity. Therefore, the butanolides that have saturated terminal or vinyl-terminal [24] were more active in anti-inflammatory than the acetylene-terminal group ones.

Comparing compounds **6**–**10**, there is no significant difference on anti-inflammatory activity of the 8-8′ linkage lignans **6** and **7**, regardless of whether there is the presence of methoxy, γ-lactone or methylene dioxide groups; instead, the symmetry lignans **8** and **9** exhibited stronger anti-inflammatory activity than asymmetric ones. The number of methoxy group and the symmetry benzene ring may play an important role to affect anti-inflammatory activity.

Comparing flavonoids **11**–**15**, there is no significant difference on anti-inflammatory activity of **11**–**13**, regardless of whether there is the presence of methoxy, *E*- or *Z*-form of the *p*-coumaroyl group, but the flavonoids, which have a rhamnoside and *p*-coumaroyl group (**11**–**13**), exhibited stronger anti-inflammatory activity than catechin (**14**) and epicatechin (**15**).

## Figures and Tables

**Table 1:** NMR data (CDCl_3_) of **1** and **6**. δ in ppm, *J* in Hz.

	1		6	

No.	δ_H_^a^	δ_C_^b^	δ_H_^c^	δ_C_^d^
1				132.4
2	175.6	6.61 (*s*)	109.3	
3	3.19 (*dd*, *J* = 6.5, 4.7)	52.7		146.5
4	4.23 (*dd*, *J* = 4.7, 4.5)	74.6		143.8
5	4.64 (*qd*, *J* = 6.5, 4.5)	78.1	6.79 (*d*, *J* = 8.0)	114.2
6	1.39 (*d*, *J* = 6.5)	13.8	6.68 (*d*, *J* = 8.0)	121.7
7	5.37 (*dd*, *J* = 15.4, 6.5)	120.9	2.62 (*dd*, *J* = 12.8, 5.0)	35.9
			2.72 (*dd*, *J* = 12.8, 8.5)	
8	5.72 (*dt*, *J* = 15.4, 7.4)	136.7	1.85 (*m*)	44.1
9	2.04 (*q*, *J* = 7.4)	32.6	3.51 (*dd*, *J* = 11.2, 6.4)	60.6
			3.77 (*dd*, *J* = 11.2, 6.4)	
10–15	1.24 (br *s*)	29.0–31.9		
16–17	1.24 (br *s*)	22.7		
18	0.86 (*t*, *J* = 6.6)	14.1		
1′				134.4
2′			6.61 (*s*)	111.4
3′				147.6
4′				145.7
5′			6.62 (*d*, *J* = 8.0)	108.1
6′			6.57 (*d*, *J* = 8.0)	121.9
7′			2.62 (*dd*, *J* = 12.8, 5.0)	35.9
			2.72 (*dd*, *J* = 12.8, 8.5)	
8′			1.85 (*m*)	44.1
9′			3.51 (*dd*, *J* = 11.2, 6.4)	60.5
			3.77 (*dd*, *J* = 11.2, 6.4)	
OCH_3_			3.82 (*s*)	55.9
OCH_2_O			5.90 (*s*)	100.8

Recorded at

a 400 MHz;

b 100 MHz;

c 500 MHz;

d 125 MHz.

**Table 2:** Cell viability and *in vitro* decrease of nitrite of LPS-stimulated production in RAW 264.7 cell activities of compound **1**–**15**.

Compound	Cytotoxicity IC_50_ (μM)	Inhibition of NO production IC_50_ (μM)
1	78.0 ± 5.1	20.1 ± 0.3
2	32.6 ± 0.5	4.1 ± 0.1
3	27.7 ± 1.6	4.5 ± 0.1
4	138.8 ± 2.8	21.7 ± 0.4
5	142.8 ± 1.9	33.4 ± 1.0
6	>292.4	196.0 ± 4.0
7	>279.3	178.8 ± 12.1
8	>239.2	49.7 ± 4.5
9	>279.3	90.4 ± 8.6
10	>400.0	311.6 ± 14.1
11	>84.5	62.5 ± 2.2
12	>86.5	67.9 ± 1.9
13	>86.5	76.9 ± 7.3
14	>517.2	413.8 ± 6.9
15	>517.2	351.7 ± 37.4
indomethacin		182.9 ± 5.5

Values are expressed as mean ± SD of three replicates.

**Table 3:** NMR data (Methanol-*d*_4_) *of***11** and **12**. δ in ppm, *J* in Hz.

	11		12	

No.	δ_H_^a^	δ_C_^b^	δ_H_^a^	δ_C_^b^
2		159.1		159.6
3		135.7		135.8
4		179.6		179.9
4a		106.1		106.1
5		158.7		158.8
6	6.22, *d*, *J* = 2.0	100.1	6.21, *d*, *J* = 2.0	100.1
7		166.2		166.1
8	6.38, *d*, *J* = 2.0	95.0	6.38, *d*, *J* = 2.0	95.0
8a		163.3		163.4
1′		124.1		122.7
2′	7.84, *d*, *J* = 8.8	132.0	7.73, *d*, *J* = 8.5	132.1
3′	7.14, *d*, *J* = 8.8	115.4	6.94, *d*, *J* = 8.5	116.6
4′		163.6		161.8
5′	7.14, *d*, *J* = 8.8	115.4	6.94, *d*, *J* = 8.5	116.6
6′	7.84, *d*, *J* = 8.8	132.0	7.73, *d*, *J* = 8.5	132.1
1″	5.62, *br s*	102.4	5.51, *d*, *J* = 1.0	102.9
2″	4.23, *br s*	71.9	4.23, *dd, J = 3.0, 1.0*	72.0
3″	3.91, *dd*, *J* = 9.7, 2.9	70.2	3.89, *dd*, *J* = 9.7, 3.0	70.3
4″	4.91, *t*, *J* = 9.7	74.9	4.90, *t*, *J* = 9.7	74.6
5″	3.18, *m*	69.8	3.28, *m*	69.9
6″	0.78, *d*, *J* = 6.3	17.8	0.78, *d*, *J* = 6.3	17.8
1‴		168.8		167.8
2‴	6.25, *d*, *J* = 16.0	115.3	5.75, *d*, *J* = 12.8	116.0
3‴	7.53, *d*, *J* = 16.0	146.8	6.87, *d*, *J* = 12.8	145.8
4‴		127.3		127.7
5‴	7.49, *d*, *J* =8.6	131.4	7.66, *d*, *J* = 8.6	134.0
6‴	6.84, *d*, *J* = 8.6	117.0	6.74, *d*, *J* = 8.6	116.0
7‴		161.4		160.3
8‴	6.84, *d*, *J* = 8.6	117.0	6.74, *d*, *J* = 8.6	116.0
9‴	7.49, *d*, *J* = 8.6	131.4	7.66, *d*, *J* = 8.6	134.0
OCH_3_	3.85, *s*	56.3		

Recorded at

a 500 MHz;

b 125 MHz.

## References

[1] Chen C.C., Lin C.F., Huang Y.L. (1995). Bioactive constituents from the flower buds and peduncles of *Lindera megaphylla*. J. Nat. Prod.

[2] Chang Y.C., Chen C.Y., Chang F.R., Wu Y.C. (2001). Alkaloids from *Lindera glauca*. J. Chin. Chem. Soc.

[3] Cheng X.L., Ma S.C., Wei F., Wang G.L., Xiao X.Y., Lin R.C. (2007). A new sesquiterpene isolated from *Lindera aggregata* (SIMS) KOSTERM. Chem. Pharm. Bull.

[4] Takamasa O., Akito N., Munehiro N., Makoto I., Li Y.M., Shinya M., Hajime M., Hisayoshi F. (2005). New sesquiterpene lactones from water extract of the root of *Lindera strychnifolia* with cytotoxicity against the human small cell cancer, SBC-3. Tetrahedron. Lett.

[5] Kouni I., Hirai A., Fukushige A., Jiang Z.H., Takashi T. (2001). New eudesmane sesquiterpenes from the root of *Lindera strychnifolia*. J. Nat. Prod.

[6] Chang S.Y., Chen M.J., Peng C.F., Chang H.S., Chen I.S. (2008). Antimycobacterial butanolides from the root of *Lindera akoensis*. Chem. Biodivers.

[7] Zhang M., Zhang C.F., Sun Q.S., Wang Z.T. (2006). Two new compounds from *Lindera chunii* Merr. Chin. Chem. Lett.

[8] Leong Y.W., Harrison L.J., Bennett G.J., Kadir A.A., Connolly J.D. (1998). A dihydrochalcone from *Lindera lucida*. Phytochemistry.

[9] Song M.C., Nigussie F., Jeong T.S., Lee C.Y., Regassa F., Markos T., Baek N.I. (2006). Phenolic compounds from the roots of *Lindera fruticosa*. J. Nat. Prod.

[10] Song M.C., Nigussie F., Yang H.J., Kim H.H., Kim J.Y., Chung D.K., Baek N.I. (2008). Phenolic glycosides from *Lindera fruticosa* root and their inhibitory activity on osteoclast differentiation. Chrm. Pharm. Bull.

[11] Wang S.Y., Lan X.Y., Xiao J.H., Yang J.C., Kao Y.T., Chang S.T. (2008). Antiinflammatory activity of *Lindera erythrocarpa* fruits. Phytother. Res.

[12] Ohno T., Takemura G., Murata I., Kagawa T., Akao S., Minatoguchi S., Fujiwara T., Fujiwara H. (2005). Water extract of the root of *Lindera strychnifolia* slows down the progression of diabetic nephropathy in *db/db* mice. Life Sci.

[13] Zhao Q., Zhao Y., Wang K. (2006). Antinociceptive and free radical scavenging activities of alkaloids isolated from *Lindera angustifolia* Chen. J. Ethnopharmacol.

[14] Department of Health, Committee on Chinese Medicine and Pharmacy (2003). The Catologue of Medicinal Plant Resourses in Taiwan.

[15] Kondo S., Mitsunaga T. (2008). Anti-inflammatory Agents Containing Butanolides. JP.

[16] Kim N.Y., Ryu J.H. (2003). Butanolides from *Machilus thunbergii* and their inhibitory activity on nitric oxide synthesis in activated macrophages. Phytother. Res.

[17] Lee S.S., Chang S.M., Chen C.H. (2001). Chemical constituents from *Alseodaphne andersonii*. J. Nat. Prod.

[18] Cheng W., Zhu C.G., Xu W.D., Fan X.N., Yang Y.C., Li Y., Cheng X.G., Wang W.J., Shi J.G. (2009). Chemical constituents of the bark of *Machilus wangchiana* and their biological activities. J. Nat. Prod.

[19] Tsai I.L., Hung C.H., Duh C.Y., Chen J.H., Lin W.Y., Chen I.S. (2001). Cytotoxic butanolides from the stem bark of formosan *Lindera communis*. Planta Med.

[20] Kaoru U., Ariko S., Masanori K., Akiru U., Takao T. (1993). Studies on differentiation-inducers from arctium fructus. Chem. Pharm. Bull.

[21] Chen C.Y., Wu T.Y., Chang F.R., Wu Y.C. (1998). Lignans and kauranes from the stems of *Annona Cherimola*. J. Chin. Chem. Soc.

[22] Cowan S., Stewart M., Abbiw D.K., Latif Z., Sarker S.D., Nash R.J. (2001). Lignans from *Strophanthus gratus*. Fitoterapia.

[23] Chang H.S., Lee S.J., Yang C.W., Chen I.S. (2010). Cytotoxic Sesquiterpenes from *Magnolia kachirachirai*. Chem. Biodivers.

[24] Walmir S.G., Massayoshi Y., Otto R.G. (1995). Benzylisoquinoline alkaloids and flavonols from *Ocotea vellosiana*. Phytochemistry.

[25] Zhao J., Zhou X.W., Chen X.B., Wang Q.X. (2009). α-Glucosidase inhibitory constituents from *Toona sinensis*. Chem. Nat. Compd.

[26] Yang C.P., Huang G.J., Huang H.C., Chen Y.C., Chang C.I., Wang S.Y., Chen I.S., Tseng Y.H., Chien S.C., Kuo Y.H. (2012). A new anti-inflammatory butanolide from the aerial part of *Lindera akoensis*. Molecules.

[27] Ha T.J., Lee J.H., Lee M.H., Lee B.W., Kwon H.S., Park C.H., Shim K.B., Kim H.T., Baek I.Y., Jang D.S. (2012). Isolation and identification of phenolic compounds from the seeds of *Perilla frutescens* (L.) and their inhibitory activities against α-glucosidase and aldose reductase. Food Chem.

[28] Geller D.A., Billiar T.R. (1998). Molecular biology of nitric oxide synthases. Cancer Metastasis Rev.

[29] Moncada S., Palmer R.M., Higgs E.A. (1991). Nitric oxide: Physiology, pathophysiology, and pharmacology. Pharmcol. Rev.

[30] Luo Y., Liu M., Dai Y., Yao X., Xia Y., Chou G., Wang Z. (2010). Norisoboldine inhibits the production of pro-inflammatory cytokines in lipopolysaccharide-stimulated RAW 264.7 cells by down-regulating the activation of MAPKs but not NF-κB. Inflammation.

